# Frugal Heart Rate Correction Method for Scalable Health and Safety Monitoring in Construction Sites

**DOI:** 10.3390/s23146464

**Published:** 2023-07-17

**Authors:** Piotr Sowiński, Kajetan Rachwał, Anastasiya Danilenka, Karolina Bogacka, Monika Kobus, Anna Dąbrowska, Andrzej Paszkiewicz, Marek Bolanowski, Maria Ganzha, Marcin Paprzycki

**Affiliations:** 1 Faculty of Mathematics and Information Science, Warsaw University of Technology, ul. Koszykowa 75, 00-662 Warsaw, Poland; krachwal@ibspan.waw.pl (K.R.); danastas@ibspan.waw.pl (A.D.); karolina.bogacka@ibspan.waw.pl (K.B.); maria.ganzha@ibspan.waw.pl (M.G.); 2 Systems Research Institute, Polish Academy of Sciences, ul. Newelska 6, 01-447 Warsaw, Poland; marcin.paprzycki@ibspan.waw.pl; 3 Department of Personal Protective Equipment, Central Institute for Labour Protection—National Research Institute, ul. Wierzbowa 48, 90-133 Lodz, Poland; mokob@ciop.lodz.pl (M.K.); andab@ciop.lodz.pl (A.D.); 4 Department of Complex Systems, Faculty of Electrical and Computer Engineering, Rzeszow University of Technology, al. Powstańców Warszawy 12, 35-959 Rzeszów, Poland; andrzejp@prz.edu.pl (A.P.); marekb@prz.edu.pl (M.B.)

**Keywords:** heart rate monitoring, frugal AI, measurement correction, occupational health and safety, IoT, scalable IoT

## Abstract

Continuous, real-time monitoring of occupational health and safety in high-risk workplaces such as construction sites can substantially improve the safety of workers. However, introducing such systems in practice is associated with a number of challenges, such as scaling up the solution while keeping its cost low. In this context, this work investigates the use of an off-the-shelf, low-cost smartwatch to detect health issues based on heart rate monitoring in a privacy-preserving manner. To improve the smartwatch’s low measurement quality, a novel, frugal machine learning method is proposed that corrects measurement errors, along with a new dataset for this task. This method’s integration with the smartwatch and the remaining parts of the health and safety monitoring system (built on the ASSIST-IoT reference architecture) are presented. This method was evaluated in a laboratory environment in terms of its accuracy, computational requirements, and frugality. With an experimentally established mean absolute error of 8.19 BPM, only 880 bytes of required memory, and a negligible impact on the performance of the device, this method meets all relevant requirements and is expected to be field-tested in the coming months. To support reproducibility and to encourage alternative approaches, the dataset, the trained model, and its implementation on the smartwatch were published under free licenses.

## 1. Introduction

The construction industry is one of the most challenging sectors in terms of ensuring workers’ safety and health. According to the Eurostat, the highest incidence of non-fatal accidents at work in 2020 was observed in construction, with 2987 such accidents per 100,000 employed persons [1]. Construction is a multi-risk work environment that, according to the categorization provided by the European Agency for Safety and Health at Work [2], includes eleven groups of risks of accidents, with slips, trips, and falls constituting the largest group. Increased fatigue in construction workers due to the intensive physical workload, work in a hot microclimate, and direct exposure to UV radiation, all contribute to a rise in the number of human errors and dangerous behaviors [3,4,5,6]. The consequence is an increased number of injuries and accidents. Therefore, special measures are needed that will provide both blue-collar workers and occupational safety and health (OSH) managers with real-time hazard monitoring and trustworthy recommendations toward improving safety and health at the construction site. In the case of physical fatigue and thermal load, heart rate (HR) is considered to be an effective indicator for early detection of potential hazards to workers’ health [7,8,9].

The rapid development of Internet of Things (IoT) technologies has enabled a fundamental paradigm shift in how OSH is approached in high-risk, dynamic environments. By leveraging the recent achievements in the areas of wearable sensors and actuators, integrated with the smart personal protective equipment (PPE) used in the workplace, it has become possible to rapidly detect hazards and manage occupational risks. This is particularly applicable to workplaces in which the environmental conditions are subject to dynamic changes that can have serious consequences for human health and life [10]. This paradigm shift consists of moving away from the traditional methods for carrying out collective risk assessments for specific groups of workers to assessment methods that allow for the determination of the level of risk individually for each worker. Furthermore, the existing periodical risk assessment approaches are replaced by the continuous monitoring of hazards in the work environment, in real or near-real time [11], and facilitate the application of time-series-oriented anomaly detection methods to monitor dangerous events [12].

### 1.1. Requirements for IoT Systems in Safety and Health Monitoring

The potential for the adoption of IoT-based systems in the construction sector for improving worker safety and health has already been noticed by several researchers. Kanan et al. [13] proposed an autonomous system that is able to monitor, locate, and warn construction workers entering danger zones. The specific issue of the safety of road repair workers was undertaken by Ma Li [14], who proposed an IoT-based system with the use of a robotic arm, aimed at preventing accidents due to thermal stress and vehicle traffic. In the paper, several types of notifications for workers were proposed, including a recommendation to drink some water, a warning of the possibility of slipping on the wet floor, as well as a “no entry” instruction, generated when too many workers are on site. Akinosho et al. [15] focused on the early detection of tiredness or fatigue, e.g., by means of a video monitoring system.

However, successfully implementing such systems in the construction sector requires overcoming several technical issues, such as scalability of the solution, energy efficiency, reliability of data, ease of implementation, maintaining workers’ privacy, and trustworthiness. The accuracy of the data and of the models based on them may significantly influence the reliability and overall usefulness of the system’s recommendations. In safety-related applications, predictions cannot have a high false positive rate of alarms, so as not to distract the user, creating a hazard by themselves. IoT systems often use wearable devices to monitor various physiological parameters of workers, which constitutes a particular challenge in relation to privacy, ethics, and security [16,17]. Moreover, the construction site is a very demanding and rapidly changing work environment with harsh working conditions. The presence of a large number of power tools and heavy machinery causes noticeable electromagnetic interference. Another challenge is ensuring the continuity of real-time data flow with minimal latency to allow the IoT system to respond to hazards reliably and quickly. Akinosho et al. [15] also draw attention to challenges such as the black-box nature of machine learning solutions and the cost of adopting these techniques.

The problem of high costs of wearable safety devices, seen as a key barrier to their adoption in the construction sector, was also indicated by Ibrahim et al. [18]. At the same time, a higher accuracy of the measured data is often associated with the considerably higher costs of wearable devices. Therefore, in relation to predicting physical fatigue, Anwer et al. [19] highlighted the need to compare the measurements obtained from wearable sensors with those obtained using a professional apparatus to refine the measurements delivered by the wearables.

The presented requirements for IoT systems in safety and health monitoring are not easy to address and require a system-wide approach to the design of the solution. Robust tools are needed for real-time processing of the data in a secure, privacy-aware, and performant manner. Here, the ASSIST-IoT reference architecture [20] aims to provide the necessary groundwork for building scalable, intelligent systems that span the continuum from the cloud to the edge and to the IoT devices. It was specifically designed to tackle the tough requirements for building next-generation IoT systems. One of the key concepts of ASSIST-IoT is the *enabler*, which is a set of encapsulated software or hardware components that, together, provides a set of well-defined functionalities. Some enablers are specifically designed to address the aforementioned requirements; for example, resilient and low-latency data routing on the edge is delivered by the Edge Data Broker enabler. ASSIST-IoT also provides rugged hardware components that can be used in the construction environment, namely the Gateway Edge Node (GWEN) and the location tags using Ultra WideBand (UWB) communication. The GWEN can be used for processing data with low latency on the edge and for interfacing with other devices. The UWB location tags provide real-time location information in indoor and outdoor environments.

### 1.2. Problem Statement

**Figure 1:**
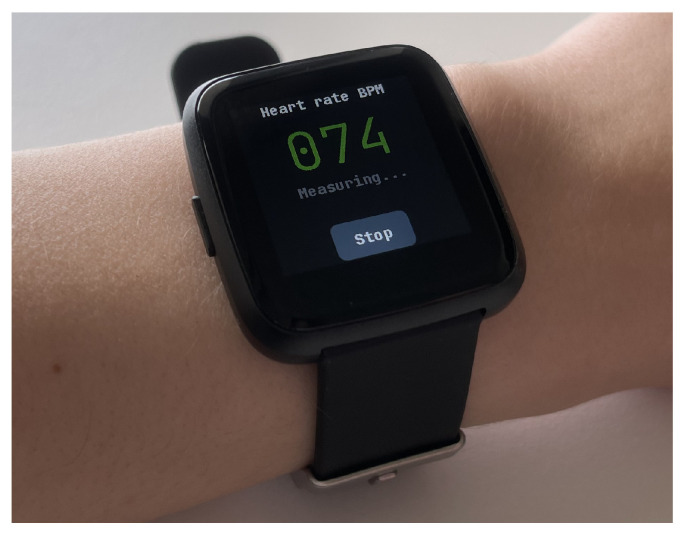
The PineTime smartwatch measuring the user’s heart rate.

According to our recent findings [21], the use of a low-cost, off-the-shelf heart rate monitoring smartwatch (the PineTime, see Figure 1) to predict fatigue and thermal stress should be possible. However, it would require implementing additional algorithms to increase the reliability of the collected heart rate measurements. Specifically, it was observed that the sensor was susceptible to various anomalies (i.e., sudden peaks in measurement were present) and that the quality of the readings depended on the dynamics of the user’s movements. The established inadequate quality of the heart rate measurement can be partially associated with the low cost of the device. However, this low cost also makes the solution scalable (devices for all construction workers can be purchased), which is a crucial requirement for this use case. Therefore, it would be beneficial if the quality of the measurement could be increased by employing *improved software* while keeping the hardware unchanged.

Therefore, the objective of this work is to improve the quality of heart rate measurements by employing a machine learning model tasked with correcting the sensor’s readings. The model will take as its input a time series of the original heart rate measurement and the readings from the smartwatch’s acceleration sensor, as context information. The output of the model will be the corrected heart rate of the user (a regression problem). Here, the mean absolute error (MAE) of the model must be no higher than 10 BPM to allow for reliable monitoring of the user’s health and safety. In the final application, the overall trend of the user’s heart rate will be used to determine whether it is consistently higher or lower than a certain threshold (e.g., higher than 90 BPM at rest [9]), which may indicate a risk to the worker’s health [7,8]. The 10 BPM target was chosen as it makes such a health risk assessment viable, at least on a basic level. Obviously, the method should be as accurate as possible, and the certainty of the prediction will depend on its accuracy. In the end application, the confidence of the result should be estimated, to allow for its responsible use.

It has to be stressed that the model needs to be integrated with the smartwatch which has very limited compute capabilities: 64 kB of RAM and a low-power ARM^®^ Cortex^®^-M4 processor running at 64 MHz (see detailed hardware specifications: https://wiki.pine64.org/wiki/PineTime#Specifications, accessed on 10 July 2023). Most of the device’s resources are already used by its real-time operating system (RTOS), which uses ~54 kB of RAM. The smartwatch also includes the HRS3300 photoplethysmographic (PPG) heart rate sensor. The limited resources place strict requirements on the size and complexity of the proposed method. Furthermore, the smartwatch must have a long battery life, a fact that also restricts the compute complexity of the solution. Both of these requirements call for a lightweight, *frugal* approach. Frugal machine learning emphasizes working with scarce data and computational resources. This frugality can be estimated with a measure chosen depending on the specific application, which embodies the accuracy/resource use trade-off [22]. In the presented use case, the most important source of frugality is the limited amount of memory and computing resources that can be used by the model.

The presented formulation of the problem is directly motivated by the characteristics of the selected hardware platform, the low-cost PineTime smartwatch. Moreover, the solution is intended to be general, requiring only readings from two very common types of sensors, included in many types of smartwatches (an accelerometer and a heart rate sensor), making it potentially applicable to a wide variety of existing devices. Note that this is a vital aspect of the developed system, as it allows one to switch hardware vendors if the need arises, making the developed solution sustainable in the long term.

Here, it should be noted that PineTime, in contrast to many other commercial smartwatches or health trackers, does not connect to a proprietary service to store or process the user’s data. This allows for all data processing to occur locally, increasing the user’s privacy and facilitating data sovereignty.

### 1.3. Structure of This Contribution

This work is organized as follows. A review of the related literature is presented in Section 2. Section 3 introduces the used dataset, its collection procedure, and the applied post-processing steps. Next, in Section 4, the proposed method is presented, including the data sampling strategies, the structure of the machine learning model, the tuning of the model’s architecture, the implementation in the smartwatch, and the integration of the model with the rest of the OSH monitoring system. Section 5 provides a thorough evaluation of the solution’s accuracy, performance, and frugality. The significance of the obtained results and their implications are discussed in Section 6, followed by the concluding remarks in Section 7.

## 2. Related Works

The PPG measurement method has been widely discussed in the literature. Of particular interest are the sources of errors in the measurements. In one evaluation of multiple consumer-grade smartwatches with heart rate sensors [23], the accuracy of measurement was found to depend on the type of performed activity. Specifically, walking yielded a higher error than running or cycling. Moreover, more intensive variants of the same activity resulted in a higher error. In a different study [24], the authors found the measurement error to be significantly higher when the user is performing movements. Particularly detrimental were rhythmic movements, interfering with the cardiovascular signal, which is also periodic. In an overview article [25], it was noted that PPG sensor measurements are affected by body movements and the contact force between the sensor and the skin [26]. Colvonen et al. [27] reported that the PPG measurement can be affected by the user’s skin tone. However, this subject is heavily disputed [28] with no clear scientific consensus [29].

To the best of the authors’ knowledge, this contribution is the first attempt at addressing the issue of correcting the measurements of inexpensive, consumer-grade heart rate sensors. The closest problem widely discussed in the literature is the estimation of heart rate using the raw PPG signals collected directly from the sensor. These works largely focus on laboratory environments and rarely discuss implementing the algorithms in real health trackers, or smartwatches. Over the years, numerous approaches have been proposed for this problem [30,31], spanning from adaptive filters to deep neural networks. Here, a large fraction of the research is focused on alleviating the effects of *motion artifacts*, i.e., the interference resulting from the user’s movements [32,33,34]. The dominant approach is to use an additional sensor (e.g., an accelerometer) and apply its measurements to filter out the motion artifacts from the PPG signal. However, alternative approaches have also been tried, such as using two PPG sensors.

Deep PPG [35], which uses a deep convolutional network, is a particularly influential work that takes as its input the PPG and the accelerometer signals transformed into spectra using the fast Fourier transform. The resulting model is very large, with 8.5 million parameters. The authors also proposed a constrained version of the model for embedded devices with only 26 thousand parameters. However, they have not integrated this solution within an actual embedded device. This is in contrast to Tiny-HR [36], which is a deep learning method integrated with an ESP32 microcontroller. The complete machine learning pipeline was reduced with TensorFlow Lite [37] to fit in 39 kB of memory. However, the work can be seen only as a proof of concept, as the heart rate estimation algorithm was the only workload running on the embedded device. In reality, a smartwatch or a health tracker will be running a full real-time operating system, together with multiple heterogeneous applications, making the integration considerably more challenging.

Only one work with a similar problem statement to the one presented above could be found [38]. Here, the authors focused on correcting the errors in the data collected with a chest-mounted heart rate monitor. The solution used a simple statistical analysis and hand-written rules for cleaning the data. However, the discussed algorithm is very use-case specific, making several strong assumptions about the device and the intended use of the collected data. Thus, it is not applicable to the problem presented here.

In summary, although no directly comparable works can be found in the literature, the past research does provide useful insights into the possible approaches to the problem. Specifically, it was found that accelerometers are often used as an additional data source for reducing errors in PPG sensors. Moreover, neural networks have been successfully used for similar tasks.

## 3. Dataset

To address the stated problem, a new training, testing, and validation dataset was needed. The dataset had to include measurements collected from the actual PineTime smartwatch (which was the designated target device) and from a laboratory-grade heart rate monitoring device. The first was needed to obtain a set of measurements with errors typical to the specific device. The second was needed to provide a high-quality reference (i.e., the regression target variable).

### 3.1. Data Collection

**Figure 2:**
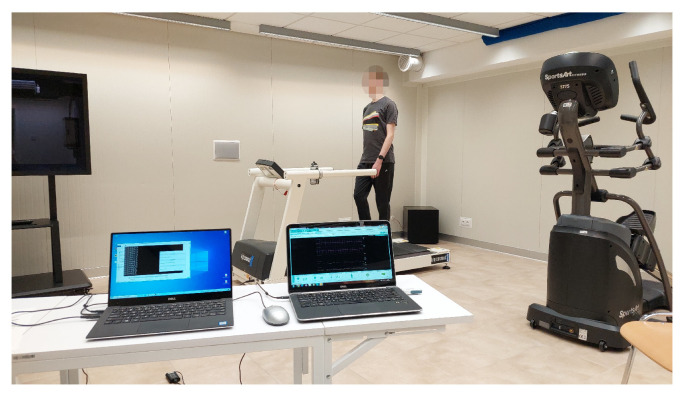
Test volunteer walking on a treadmill.

**Figure 3:**
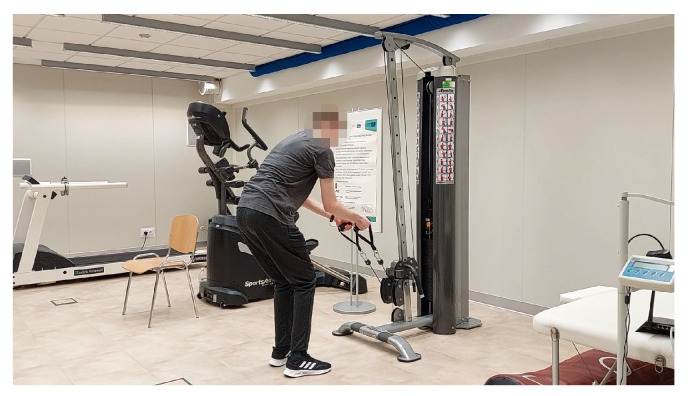
Test volunteer during upper lift exercises.

The data collection was carried out with the involvement of six volunteers, in controlled environmental conditions, in the Research and Demonstration Laboratory SMART PPE TESTLAB on the premises of CIOP-PIB. The study participants included three women and three men. The average age, height, and weight of the participants were 32.17 ± 6.11, (174.50 ± 10.99) cm, and (67.40 ± 11.73) kg, respectively. The tests were carried out in an environment with an ambient temperature of 25 °C and a relative humidity of 65%. The research procedure involved performing various physical activities, such as walking on a treadmill at 5 km/h and 3 km/h (Figure 2), as well as exercises using the upper lift (Figure 3). The exact test procedure is presented in Table 1. The test procedure was planned so as to include exercises involving hand movements to be able to analyze the impact of hand movement on the correctness of the smartwatch’s indications.

During the data collection, the heart rate of the participants was measured using both target devices. One of them was the PineTime smartwatch (Figure 1), which measures heart rate from the wrist, using the PPG method. In addition, the data from the smartwatch’s accelerometer were collected. Measurements were sent wirelessly via Bluetooth Low Energy (BLE) to a computer and saved to a file using custom laboratory software. In order to obtain the reference target value, the heart rate was additionally measured using the Equvital system eq02+ LifeMonitor. The Equvital device measures the heart rate using ECG electrodes built into the vest. This system also measures other physiological parameters, such as skin temperature and respiratory rate. Data from the system was sent wirelessly to a computer to be monitored in real time and saved to a file using the eqView Professional software.

Participation in the research was voluntary. Before the research, all participants were thoroughly informed about the test procedure and its purpose. In addition, each participant was familiarized with an Information Sheet that included information about the project, research, data collected during research, and privacy policy. Prior to the start of the experiment, the participants expressed their willingness to participate in this study by means of signing the Informed Consent Statement. All collected data were anonymized to preserve the privacy of research participants.

### 3.2. Dataset Post-Processing

The raw data collected from the Equivital and from the PineTime devices were converted to a common format—Feather [39] (based on Apache Arrow). The data from the two devices were then cleaned and aligned in the time dimension to produce a single table of measurements. As the PineTime collected heart rate measurements more often than Equivital, the alignment was completed on a nearest temporal match basis, taking the PineTime measurements as the primary time series. In the final table, each pair of heart rate measurements (from PineTime and Equivital) is associated with a series of acceleration measurements from PineTime, collected just before the given heart rate value measurement. The original timestamps from both devices were kept for reference. The selected data format was chosen to be convenient for machine learning tasks. However, the train/validation/test split is not included in the dataset, as the number of samples depends on the sampling algorithm that is used. The sampling method is detailed in Section 4.1, along with the dataset split used in this study.

**Figure 4:**
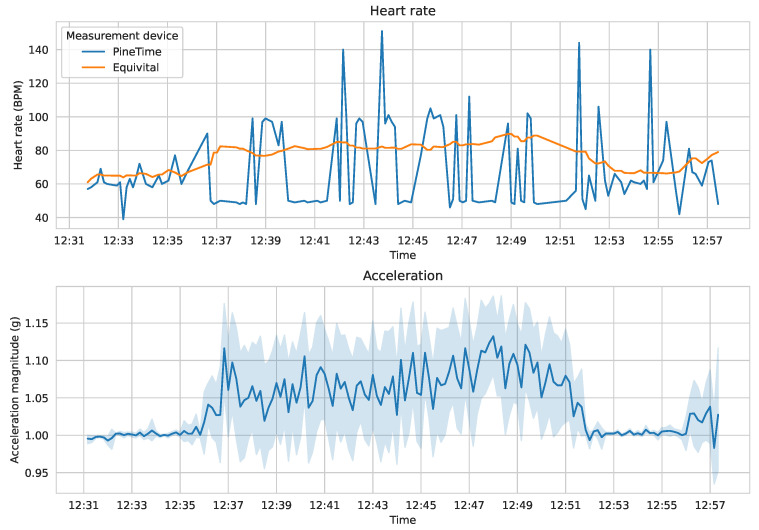
A fragment of the collected heart rate and acceleration data (study participant 1).

Figure 4 presents a fragment of the collected data with the three-axis acceleration data summarized as an overall acceleration magnitude. The errors in the measurements from the PineTime device are clearly visible, as are the differences between the activities performed (for instance, the subject was walking at 5 km/h between 12:36 and 12:51, took a break, and then started walking again at 12:56).

### 3.3. Dataset Summary

The dataset contains a total of 510 min of usable laboratory data collected from six participants. The heart rate data from the Equivital device was collected every 15 s. The heart rate data from PineTime was collected on average every 12 s. Finally, the acceleration data from PineTime was collected on average every 0.25 s.

The raw and processed data were published on Zenodo under a permissive license (CC BY 4.0) [40]. These can be used to reproduce the results presented in this contribution or to attempt alternative approaches to the same problem. The raw dataset also includes additional data collected from the Equivital device (such as thoracic motions, acceleration, and heart interbeat measurements) that were not used in this study but may be of value in other future research. Relevant anonymized laboratory notes, including the study protocol for each participant, are also included.

## 4. Proposed Approach

This section presents the machine learning approach, designed for the heart rate measurement correction problem. First, the data sampling procedure is presented. Second, the machine learning model’s architecture, training process, and final characteristics are described. Then, the model’s implementation on the smartwatch is detailed, including its integration with the device’s RTOS. Finally, the overall architecture of the solution is presented, explaining the context in which the proposed method is to be used.

### 4.1. Data Sampling

On the PineTime smartwatch, new heart rate readings are obtained on average only every 12 s, while the acceleration readings are much faster at around 4 Hz in the dataset (the smartwatch itself can collect acceleration data with a higher frequency, but the entirety of these data could not be transmitted due to bandwidth limitations). Bearing in mind the limited processing capabilities of the smartwatch, and the need to conserve its battery, it would not be feasible to perform inference every time a new acceleration value is read. Therefore, inference is only performed whenever a new heart rate value is read from the sensor, while the past acceleration readings are downsampled and fed into the model.

The following sampling method was used: let *A*, |A|=n be the set of acceleration magnitude values recorded by the accelerometer in the time window between two heart rate measurements ($h_{i-1}$ and $h_{i}$). Then, $h_{i}$ is associated with three statistical parameters of *A*—its median ($a_{i}^{med}$), maximum ($a_{i}^{max}$), and interquartile range ($a_{i}^{iqr}$). These three parameters were chosen primarily due to their very low computational requirements; they can all be obtained by sorting *A* and then applying a few O(1) operations (indexing). Secondly, these parameters intuitively capture multiple characteristics of the acceleration signal—its most common values ($a_{i}^{med}$), extremes ($a_{i}^{max}$), and variability ($a_{i}^{iqr}$).

The sampled values can then be used to build a feature vector for the machine learning model. The model may use a sliding window of past *k* acceleration samples and *m* heart rate samples as its input to obtain the necessary context. The vector can be expressed as:

(1) $$v=\langle h_{1},\ldots ,h_{m},a_{1}^{med},\ldots ,a_{k}^{med},a_{1}^{max},\ldots ,a_{k}^{max},a_{1}^{iqr},\ldots ,a_{k}^{iqr}\rangle$$

and has a length of |v|=m+3k. The parameters *m* and *k* can be adjusted independently with higher values increasing the memory and processing time requirements of the solution.

### 4.2. Machine Learning Model

The selection of the model’s architecture was guided by the use-case-driven constraints. Specifically, the final model was limited to having at most 100 parameters, due to computational and energy consumption limitations (see the explanation in Section 1). Consequently, the model was composed of fully connected layers only, resulting in a compact dense neural network (DNN). To determine the number and dimensions of the layers, a set of experiments was carried out during the architecture tuning phase (described in Section 4.3). The hidden layers use the rectified linear unit (ReLU) activation [41], which is formulated as follows:

(2) Relu(x)=max(0,x).

The output layer uses the linear activation function. These choices were motivated by the very low computational requirements of both activation functions and the high effectiveness of the ReLU activation reported in practice [42]. The inputs to the network were normalized, and the target value was set to the heart rate value, as measured by the Equivital device.

The model was implemented using the TensorFlow framework [43] and the Keras API [44]. During training the Adam optimizer was used [45] with a starting learning rate of 0.001. As the task being solved is a regression problem, the mean squared error (MSE) loss function was chosen:

(3) $$\mathrm{MSE}\left(y,\hat{y}\right)=\frac{\sum_{i=0}^{N-1}{\left(y_{i}-{\hat{y}}_{i}\right)}^{2}}{N}.$$

To mitigate possible overfitting on the limited training dataset, L2 regularization was applied to the kernels of the layers, using a regularization factor of 0.01. The batch size for the training was set to 16. Train/validation/test data split was 70/10/20, respectively. To avoid data leakage, the sample windows were picked in such a way that there are no overlapping regions between the train, validation, and test datasets.

### 4.3. Neural Architecture Tuning

Due to the small target size of the final network, architecture tuning was performed to find the optimal number of additional hidden layers and the number of units in every hidden layer. The input data size (i.e., the sampling strategy) was also a part of the tuning procedure, aimed at finding the optimal window sizes for both the heart rate and the acceleration data. The following parameters were examined during tuning:

- Size of heart rate window: [1, 3, 5, 7];
- Size of acceleration window: [1, 3, 5];
- Number of units in the first hidden layer: [1–6];
- Number of additional hidden layers: [0–2];
- Number of units in the additional hidden layers: [1–4].

The Keras Tuner library [46] was used for conducting the grid search as it provides a convenient implementation of parameter search and utilities for aggregating performance metrics. Each unique parameter set was evaluated five times, with different random starting weights, to reduce the variance of the results obtained on a specific set of parameters. In total, 648 different neural network architectures were tried and evaluated using the mean absolute error (MAE) metric, defined as:

(4) $$\mathrm{MAE}\left(y,\hat{y}\right)=\frac{\sum_{i=0}^{N-1}\left|y_{i}-{\hat{y}}_{i}\right|}{N}.$$

**Figure 5:**
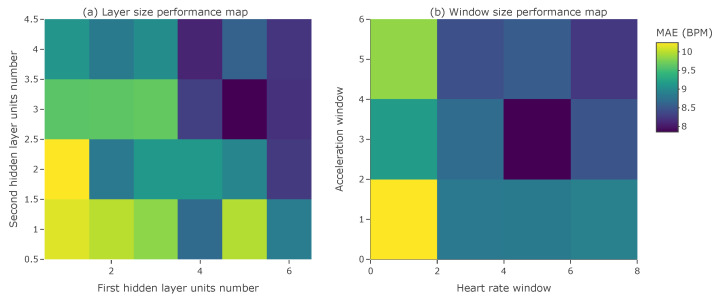
(**a**) Hidden layers size tuning results. (**b**) Heart rate and acceleration windows size tuning results.

Figure 5 illustrates the performance grids for two pairs of tuned parameters with respect to the MAE score on the validation dataset. The grids show the location of the model with one of the lowest validation MAE scores. They also indicate that more complex models with more input data tend to achieve a better performance.

**Figure 6:**
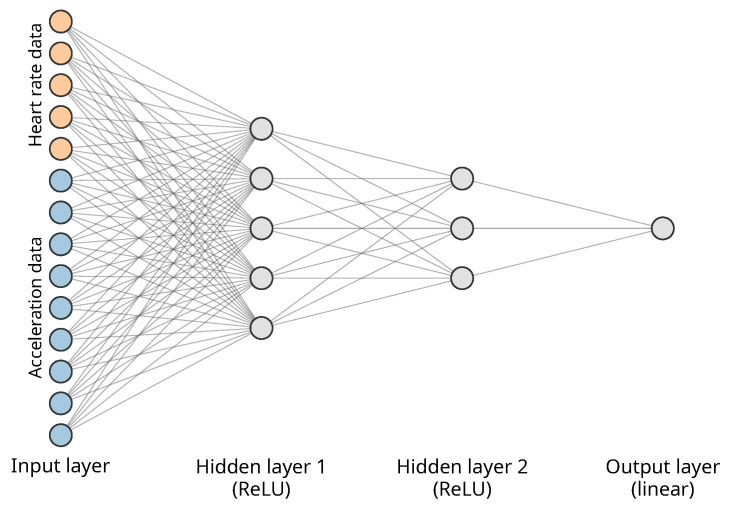
The final neural network selected during neural architecture tuning.

The final choice of parameters was dictated by the model size, as related to the best MAE. As a consequence, the best model has 97 parameters in total, with the first hidden layer having five units, and the second hidden layer having three units. A heart rate window of size 5 and an acceleration window of size 3 were also selected based on the defined selection multicriteria. The final model is presented in Figure 6. The model’s weights were published in Zenodo [47]. This final model was then used in the implementation in the smartwatch and the evaluation in Section 5.

### 4.4. Implementation

The PineTime smartwatch comes with an open-source real-time operating system (RTOS) written in C++, called InfiniTime. The RTOS provides features such as a graphical user interface (GUI), sensor drivers, and BLE connectivity. The final trained model was integrated into the RTOS by rewriting the original service for measuring the user’s heart rate, offering seamless compatibility with the original BLE service for obtaining the heart rate and with the existing GUI for presenting the heart rate to the user.

To deploy the model in the smartwatch, the model saved in the Keras .h5 file was converted to C code, using the keras2c library [48]. The generated code is minimal with no need for a full runtime. The library implements in an optimized manner the functions needed to perform the inference of Keras models. It includes representations of dense and convolutional layers, different activation functions, and utilities such as matrix multiplication. A large portion of these library functions were not needed for the built model. Therefore, to minimize the smartwatch’s resource consumption, only the required subset of the library was integrated into the RTOS.

To further minimize the performance impact of the method on the smartwatch’s performance, a number of optimizations were applied to the routines for the input data preprocessing (data sampling). The acceleration data are inserted into a buffer using insertion sort, which causes the insertion operation to be O(n) but removes the need to run any sorting algorithm afterward. The standard deviation and the mean values, needed for normalizing the inputs, were precomputed using the training data. The standard deviation values were also pre-inverted, to avoid having to perform floating point division during runtime, as this is an expensive operation on the smartwatch’s processor (ARM^®^ Cortex^®^-M4). Instead, only multiplication is needed, which is a much faster operation. Finally, to minimize the use of the smartwatch’s RAM, rolling buffers were used to store the already preprocessed data. The integrated method has a constant size in memory, avoiding dynamic memory allocation, which would introduce additional overhead.

The modified RTOS source code and the built images are available in Zenodo [47]. The modified RTOS is fully functional and can be uploaded to any off-the-shelf PineTime device.

### 4.5. Overall Solution Architecture

**Figure 7:**
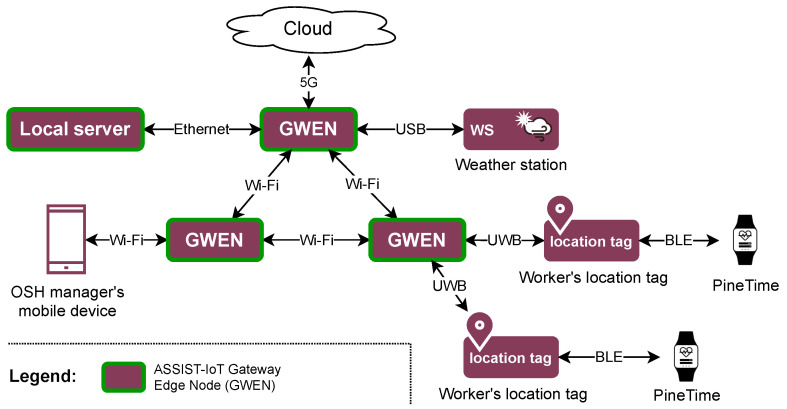
Overall hardware architecture of the solution. The network connections are represented in a simplified manner for clarity. The number of devices varies depending on the specific deployment.

**Figure 8:**
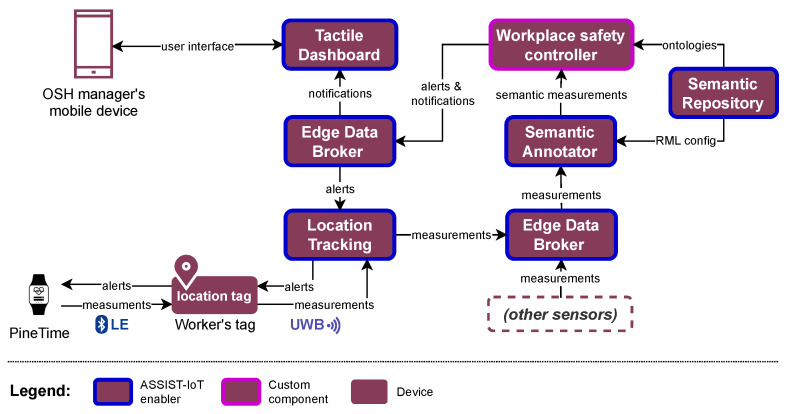
Overall software architecture of the solution in the context of the use case.

The smartwatch is only a single element of the overall architecture of the solution needed to deliver the use case. Figure 7 and Figure 8 present an overview of the hardware and software layers of the integrated solution that utilizes the ASSIST-IoT reference architecture [20]. Starting with the hardware layer, the smartwatch of each worker communicates via BLE with the worker’s location tag, which, in addition to tracking the worker’s location also serves as a wireless communication relay. The location tag connects to the nearest ASSIST-IoT Gateway Edge Node (GWEN) [20] via UWB. In this architecture, the GWENs are the main providers of compute capability, hosting the ASSIST-IoT enablers and other software components. They are connected to each other and a local server via Wi-Fi or Ethernet. The hardware diagram includes also a mobile device of the OSH manager. The manager uses it to monitor the safety of the construction site. Finally, the system includes a weather station, which collects environmental measurements that are used as context in the decision-making processes.

Regarding the software layer (Figure 8), the setup is comprised a mix of embedded software, ASSIST-IoT enablers [20], and custom components. The enablers and custom components run in the virtualized environment of ASSIST-IoT, using K3s (a lightweight distribution of Kubernetes [49]). The workloads are run as close to the worker as possible (in the GWENs on the edge). This allows for preservation of the network bandwidth. Moreover, this approach provides an additional layer of privacy for the workers, as the data are not transmitted to or stored in a central location.

The information flows from the smartwatch, which measures the acceleration and the user’s heart rate and then infers the corrected heart rate value locally. This measurement is then relayed to the location tag and the Location Tracking enabler, which is responsible for interfacing with the UWB network. The Location Tracking enabler outputs the measurements as an MQTT stream that is routed by the Edge Data Broker enabler to the Semantic Annotation enabler. The Semantic Annotation enabler annotates non-semantic data (e.g., JSON files) into semantic knowledge graphs (in RDF [50]), using the RDF Mapping Language (RML) [51]. The semantic information from various sensors is integrated and processed in the Workplace safety controller, which makes the appropriate decisions about the safety of the workers (e.g., assessing if the worker is in danger of a heat stroke). The Semantic Repository enabler plays a supporting role here by serving as a central “nexus” for storing data models used by the system, such as RDF ontologies or RML mappings. If needed, the Workplace safety controller issues alerts to workers and notifications to the OSH manager to warn them about an OSH hazard. The alerts and notifications are routed through the Edge Data Broker enabler to the appropriate recipients in the network. The worker alerts are sent back via the Location Tracking enabler and the location tag to the smartwatch. The OSH manager’s notifications, informing them only about immediate hazards to the worker’s health, are displayed on their mobile device, with the help of the Tactile Dashboard enabler. For obvious privacy reasons, the worker’s heart rate patterns are not stored permanently, and the OSH manager cannot view them. The system makes only the necessary information available to the OSH manager.

## 5. Experimental Results

This section offers an in-depth evaluation of the proposed solution, in terms of its prediction quality, performance characteristics, and frugality.

### 5.1. Prediction Quality

**Figure 9:**
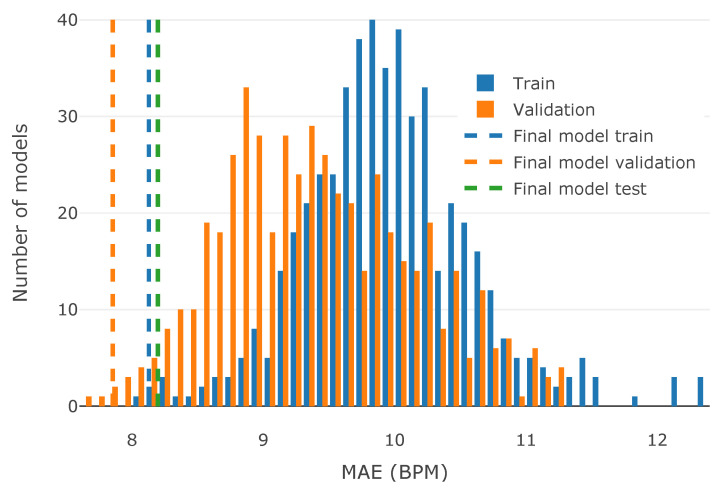
Histograms for train and validation MAE for models explored during the tuning phase. Final model’s results are indicated with dashed vertical lines.

The limited size of the collected dataset imposed restrictions on how the final model should be chosen among those that were trained (see Section 4.3). To better assess the generalization capability of the trained models, their performance was evaluated on both the training and the validation datasets. Histograms of both training and validation MAE were explored to assess the impact of the small dataset on the models’ performances (Figure 9). Each histogram represents the distribution of MAE for a given constant dataset, across all models considered during the tuning phase. This illustrates how many among the examined models fell into a particular MAE range.

It can be observed that the validation MAE is usually lower than the training MAE, which may be a sign of an insufficient dataset size. It is also apparent that the final chosen model has low MAEs for both the train and the validation datasets. The final model’s performance is presented as dashed lines of respective colors in Figure 9, illustrating that it is one of the best-performing models. Additionally, the difference between the final model’s training and validation performance is only 0.27, whereas the median difference between the training and the validation performance, across all presented models, is 0.65. In the presence of limited source data, the balanced performance on both the training and the validation subsets may better represent the generalization capability of the resulting model. This can also be seen as another tool for detecting overfitting while training the model on a small dataset.

After choosing the final model, the test dataset was used to assess the performance of the model on previously unseen data (green dashed line in Figure 9). The test dataset does not have a respective histogram, due to its absence during the tuning stage; it was used exclusively for the final model performance estimation. The resulting test MAE is **8.19 BPM**, while the train and validation MAEs are 7.85 and 8.12, respectively. The final model has a low error on all three datasets, and the maximum difference between the model’s performance on the train, validation, and test datasets is 0.34. This shows that the model’s performance on previously unseen data closely aligns with the performance observed during the model tuning phase. The final MAE of 8.19 BPM is also lower than the target MAE of 10 BPM, as required by the use case.

**Figure 10:**
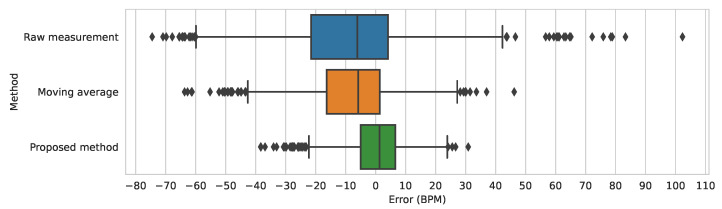
The errors of the raw measurement, the moving average, and the proposed method, in relation to the ground truth, on the test dataset. The boxes show the quartiles of the error distribution. The whiskers show the extent of the distribution, except the outliers, indicated with diamonds.

**Figure 11:**
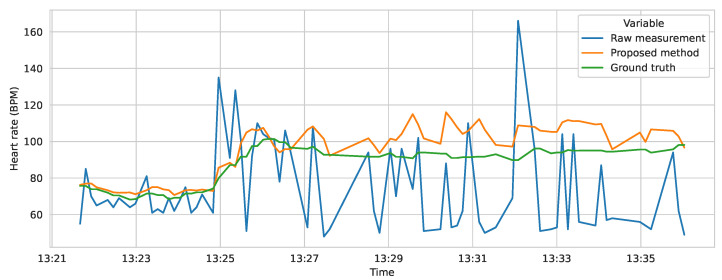
Predictions of the model compared with the ground truth (data from the Equivital device) and the raw measurements from the smartwatch’s sensor. Data from the validation and test datasets was used for this example (study participant 4).

The final model was also compared on the test dataset against two baselines—the simple moving average, which takes the mean of the last five heart rate measurements, and the raw, unmodified measurement. In the comparison, MAE and MSE (mean squared error) metrics were used. The results are presented in Table 2, showing that the proposed approach is significantly better than the moving average baseline. Figure 10 shows the distribution of errors in the raw measurement, the moving average, and the proposed method. Additionally, Figure 11 presents an example prediction of the model, compared to the ground truth and the raw measurements.

### 5.2. Performance Characteristics

The impact of the implemented method on the smartwatch’s performance was evaluated by measuring its memory consumption and CPU usage. The memory consumption is summarized in Table 3. Overall, the RAM usage of the method does not exceed 1 kB (out of 64 kB available), ensuring that enough memory is available for other functionalities of the smartwatch.

The time required to preprocess the data and perform inference was measured over 500 samples. The smartwatch measures time using an onboard counter-based clock, which ticks at a frequency of 1024 Hz. This is the most precise measurement of execution time available programmatically on this device. The mean processing time over the 500 measurement samples is 0.184 smartwatch ticks (σ=0.431), or approximately 0.18 ms. Considering that heart rate measurements are collected only every 12 s, the model’s impact on the performance of the smartwatch is negligible.

### 5.3. Frugality Score

In recent years, the term “frugality” has been very influential in machine learning research, due to the popularization of paradigms such as TinyML [52], which strive to adapt machine learning to edge and IoT environments. However, there have been no universally accepted metrics of model frugality that would consider both its resource consumption and capabilities. The lack of such a metric impedes easy comparison with other methods, developed for the specific problem. In order to promote open and reproducible research in frugal machine learning, a variant of the frugality score proposed by Evchenko et al. [22] was computed for the final model. Several changes were applied to the original definition of the score, to adapt it to the problem presented in this work. Firstly, resource consumption is expressed in a form that considers both memory and CPU usage. This is a variant of the RAM-Hours metric [22,53]. Secondly, the original performance metric of AUC is replaced with mean absolute error, as AUC cannot be used to assess a model’s performance in regression problems. The final equation used to compute the frugality score, forming the frugality curve of the final model, is as follows:

(5) $${\mathrm{Frug}}_{\mathrm{DNN}}^{\mathrm{smartwatch}}={\mathrm{MAE}}_{\mathrm{test}}-\frac{w}{1+\frac{1}{U_{\mathrm{RAM}}*T_{\mathrm{CPU}}}}$$

**Figure 12:**
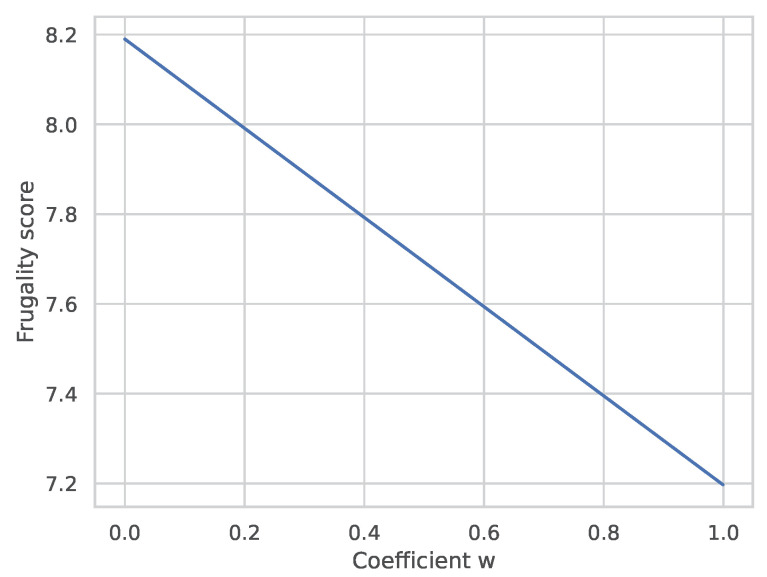
The frugality curve of the final model.

Here, $MAE_{test}$ is the mean absolute error computed for the model on the test dataset. The values of the RAM usage $U_{RAM}$ and the CPU time $T_{CPU}$ are represented in bytes and milliseconds, respectively. The coefficient *w* expresses the importance of resource frugality in the final value of the score, with 0 indicating no resource scarcity and 1 indicating extremely scarce resources. The frugality score was plotted for different values of *w* (Figure 12), forming the frugality curve. The RAM and CPU time consumption used to compute the frugality curve were measured on the PineTime smartwatch. The case of RAM consumption includes only the memory used by the method. The curve can be used to compare the frugality score of the method described in this work with those of other methods. The scores may be analyzed for a given level of resource frugality needed for the problem indicated in the form of the *w* coefficient. The authors encourage modification of this frugality score and the reuse of the frugality curve for research purposes.

## 6. Discussion

The presented method of correcting heart rate measurements in an off-the-shelf, low-cost smartwatch is, to the best of the authors’ knowledge, the first attempt at solving this particular problem. The method was found to meet the requirements of the construction site use case. Firstly, the target of prediction performance (MAE of 10 BPM) was met, with a final MAE of 8.19 BPM. Secondly, the computational requirements of the method are very low, with only 880 bytes of RAM needed for the whole, integrated method, and a negligible impact on CPU time. This indicates that it may be possible to develop even more complex methods for correcting heart rate measurements in the smartwatch, possibly by using longer feature vectors or by increasing the neural network’s size. The prediction performance results (see Section 5.1) clearly indicate that the method would benefit from a larger dataset.

In the context of the real-life use case, the method fulfills an important need and can be expected to contribute significantly to improving the safety of workers in construction sites and other demanding environments. Here, it is worth noting that the proposed system architecture (Section 4.5) is a holistic solution for detecting health-related hazards to workers, exemplifying how the ASSIST-IoT reference architecture may be used to tackle a demanding problem using a mix of cloud, edge, and IoT resources. It also illustrates how ASSIST-IoT enablers can be used as blocks of reusable features that can be easily added to a system.

It is important to stress that significant efforts were made to ensure the reproducibility of this study and to invite future research contributions to this topic. First, the collected dataset, trained model weights, and the method’s implementation were open-sourced and made available under free licenses. Second, the obtained frugality curve (Section 5.3) allows for making reliable comparisons of the accuracy/computational requirements trade-off between different methods attempting to tackle this problem.

Nevertheless, this study has some limitations that must be acknowledged. The dataset size could be increased to make it more representative. People of diverse height, build, and skin tone should be included. In particular, some studies found darker skin tones to negatively influence the quality of measurement using the PPG method [29]. It is not known whether this effect applies to the sensor in the PineTime smartwatch, and it remains a subject for future research. Furthermore, the performance of the method was evaluated in a laboratory setting, whereas a construction site is a much harsher environment, with excessive vibrations, electromagnetic interference, dust, and moisture. This may affect the results significantly, and, thus, a real-life study of the system’s performance should be conducted.

## 7. Concluding Remarks

In this work, a novel frugal method for correcting heart rate measurements is presented. The method is motivated by a real use case of monitoring health hazards to workers in an active construction site, where scalability and cost effectiveness are some of the primary concerns. Therefore, the method allows one to employ low-cost, off-the-shelf smartwatches for this task. Not only is a machine learning model presented but also its seamless integration with the smartwatch’s RTOS, making the solution readily applicable in the field. Furthermore, a design for a wider, end-to-end solution to health and safety monitoring in a construction site is presented, making use of the modular ASSIST-IoT reference architecture. The presented method was evaluated in terms of its prediction quality, impact on the smartwatch’s performance, and frugality. In all of the evaluated aspects, the method was found to meet the relevant requirements, as dictated by the use case. Even more importantly, the developed solution can easily generalize to multiple other scenarios involving workers’ health monitoring, e.g., in ports and logistics centers, factories, mines, etc.

Finally, due to the requirements of the ASSIS-IoT project, the presented method and its integration with the ASSIST-IoT reference architecture are scheduled to be field-tested in the coming months in an active construction site in Poland. The method’s usability, accuracy, and reliability will be comprehensively tested in a number of realistic scenarios.

## Figures and Tables

**Table 1:** Research procedure.

Type of Activity	Duration (min)
Break	5
Activity I—walk at a speed of 5 km/h	15
Break	5
Activity II—walk at a speed of 3 km/h	15
Break	5
Activity III—walk at a speed of 5 km/h	15
Break	5
Activity IV—upper lift exercises, dynamic movements	5
Break	5
Activity V—upper lift exercises, calm movements	5
Break	5

**Table 2:** Comparison of the proposed method’s accuracy against the baselines, on the test dataset. The error values are in BPM.

Method	MAE	MSE
Raw measurement	19.39	727.19
Moving average	13.72	337.29
Proposed method	8.19	119.30

**Table 3:** Memory usage of the method implemented on the smartwatch.

Memory Region	Total Used	Used by Method	Total Size
FLASH	420,516 B	2992 B	474,632 B
RAM	55,256 B	880 B	65,536 B

## Data Availability

The data presented in this study are openly available in Zenodo at https://doi.org/10.5281/zenodo.7963497 (accessed on 10 July 2023), reference number 7963497. The implementation of the presented method is openly available in Zenodo at https://doi.org/10.5281/zenodo.7963495 (accessed on 10 July 2023), reference number 7963495.

## Abbreviations

The following abbreviations are used in this manuscript:

UV	ultraviolet
OSH	occupational safety and health
HR	heart rate
IoT	Internet of Things
PPE	personal protective equipment
GWEN	Gateway Edge Node
UWB	Ultra WideBand
PPG	photoplethysmographic
RTOS	real-time operating system
BLE	Bluetooth Low Energy
MAE	mean absolute error
GUI	graphical user interface
RAM	random access memory
MQTT	MQ Telemetry Transport
